# Surface Formation Pathway of Nitrogen- and Sulfur-Containing Organic Compounds on Ammonium Sulfate

**DOI:** 10.1021/acs.jpca.5c00332

**Published:** 2025-03-13

**Authors:** Jie Chen, George Wandera Kisimbiri, Ivan Gladich, Nicolas Fauré, Erik S. Thomson, Robert Temperton, Zamin A. Kanji, Xiangrui Kong

**Affiliations:** † Institute for Atmospheric and Climate Science, ETH Zürich, Zurich 8092, Switzerland; ‡ Department of Chemistry and Molecular Biology, University of Gothenburg, 41390 Gothenburg, Sweden; § European Centre for Living Technology (ECLT), Dorsoduro, Calle Crosera, 30124 Venice, Italy; ∥ Qatar Environment and Energy Research Institute, Hamad Bin Khalifa University, 31110 Doha, Qatar; ⊥ MAX IV Laboratory, Lund University, 22100 Lund, Sweden

## Abstract

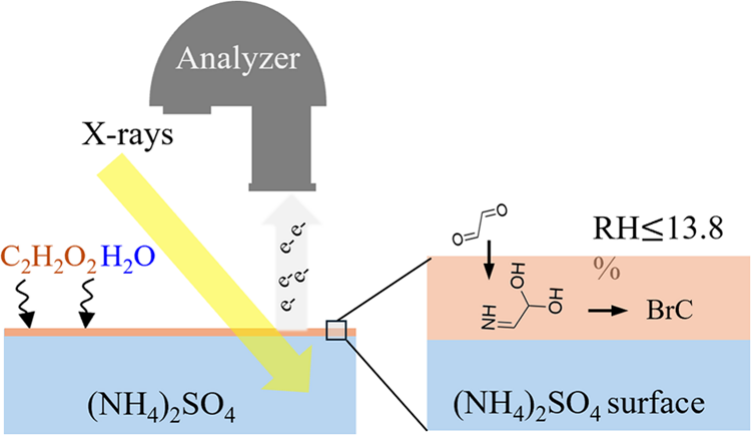

The formation of nitrogen- and sulfur-containing organic compounds (N-Org and S-Org) is important for atmospheric secondary organic aerosol (SOA) production, thereby influencing air quality and global climate. However, the mechanisms underlying N-Org and S-Org formation on aerosol particle surfaces are poorly understood due to the limited availability of surface-sensitive analytical techniques. This study investigates the surface interactions of glyoxal (GL), a known SOA precursor, with ammonium sulfate (NH_4_)_2_SO_4_, under varying relative humidity (RH) conditions, using ambient-pressure X-ray photoelectron spectroscopy (APXPS), near-edge X-ray absorption fine structure (NEXAFS) spectroscopy, and molecular dynamics (MD) simulations. N-Org species, such as imines, a key intermediate in brown carbon (BrC) formation, are identified on the (NH_4_)_2_SO_4_ surface at low RH (≤13.3%). The formed S-Org species cannot be specified due to the difficulties in distinguishing S-Org from inorganic sulfate in the XPS spectra. Elemental ratios on (NH_4_)_2_SO_4_ surface across the entire probing depth show increased S/O and N/O ratios upon GL exposure, indicating the formation of N-Org and S-Org species. NEXAFS measurements further confirm the surface changes of (NH_4_)_2_SO_4_ associated with the adsorption of GL and water. These findings provide compelling evidence of surface-driven N-Org and S-Org formation pathways, demonstrating that heterogeneous reactions on (NH_4_)_2_SO_4_ particle surfaces could be an active source of atmospheric BrC and SOA.

## 1 Introduction

Secondary organic aerosol (SOA) contributes to a significant portion of ambient aerosol in the troposphere.^1^ Despite its pivotal role in influencing air quality and global climate,^2,3^ the mechanisms underlying the formation and transformation of SOA in the atmosphere remain incompletely understood. SOA can originate from the oxidation of volatile organic compounds (VOCs) followed by the nucleation and condensation of low-volatile oxidation products.^4,5^ Alternatively, they can form through heterogeneous and multiphase reactions,^5−9^ such as oxidation of VOC products on aerosol particles or within aqueous solution droplets. Compared to SOA produced through gas-phase oxidation and nucleation, the formation pathways of SOA from heterogeneous reactions are less well understood, leading to their incomplete representation in current atmospheric models^10,11^ and discrepancies between SOA yields observed in the field and those simulated by models.^12,13^

Glyoxal (GL), a prevalent α-dicarbonyl compound originating from various biogenic and anthropogenic emissions, is recognized as a substantial precursor and tracer of SOA formation.^14,15^ GL actively participates in multiphase chemical processes,^16−18^ attributed to its strong water solubility and significant atmospheric presence. It is estimated that GL-derived SOA could account for 3 Tg year^–1^ of observed SOA.^15,19,20^ Particularly noteworthy are reactions between GL and ammonium salts, given their potential to produce light-absorbing organic aerosol (specifically brown carbon, BrC),^21^ an important contributor to aerosol–radiation interactions. Aqueous phase reactions between GL and ammonium sulfate (NH_4_)_2_SO_4_ solutions,^22,23^ as reviewed by Laskin et al.,^21^ form nitrogen-containing organic compounds with pronounced absorption at wavelengths of 207 and 280 nm. The primary BrC products identified in this reaction are imidazole-based compounds including imidazole, biimidazole (BI), and imidazole-2-carboxaldehyde (IC).^22,23^ The reactive uptake of gas-phase GL onto (NH_4_)_2_SO_4_ aerosol particles over a broad range of relative humidities (RHs) is also observed in aerosol chamber studies.^24−26^ Commonly identified products in these experiments, as detected by mass spectrometry, include imidazole-based compounds^26^ along with GL monomers/oligomers^26^ and various acids.^24,27^ In a recent chamber experiment, De Haan et al.^27^ quantified BrC formation on GL-exposed (NH_4_)_2_SO_4_ particles under an extremely dry condition (at RH = 5%). Their findings confirmed that the enhanced light absorption of SOA produced in their experiment was associated with the formation of particle-phase imidazole, pyrazine, and IC. Unlike previous studies that focused on BrC formation in wet aerosol particles^24−26^ or aqueous solutions,^22,23,25^ De Haan et al.^27^ imply the role of surface reactions in producing BrC on dry salt particles. In addition to nitrogen-containing organic compounds, the formation of organosulfate in GL-reacted (NH_4_)_2_SO_4_ aerosol was proposed by Liggio et al.^28^ They detected two peaks (CH_5_O_6_S^+^ and C_2_H_7_O_7_S^+^) using an aerosol mass spectrometer (AMS), which were assigned to fragmentation products of GL-sulfate.

Current aerosol chamber experiments on SOA formation from GL-reacted salt particles imply that in addition to aqueous phase reactions on wet aerosol particles or within solution droplets, surface reactions on dry (NH_4_)_2_SO_4_ particles may also contribute to atmospheric BrC production. However, this hypothesis has not been validated at the particle-surface level due to the absence of in situ measurements for reactions occurring on particle surfaces. Directly measuring SOA formation on particle surfaces presents a challenge owing to the rapid alteration of particle surfaces with extremely limited areas during surface reactions. To address these limitations, ambient-pressure X-ray photoelectron spectroscopy (APXPS),^29^ with its high temporal resolution and surface sensitivity, serves as a novel and powerful tool. In this work, we characterize the (NH_4_)_2_SO_4_ surface and its interaction with GL under low-RH conditions (≤13.3%) using a synchrotron-based APXPS system together with electron detection near-edge absorption fine structure (NEXAFS) spectroscopy.

## 2 Methodology

### 2.1 APXPS

Measurements were performed at the HIPPIE beamline^30^ at the Swedish National Synchrotron Radiation Facility MAX IV Laboratory. HIPPIE can provide photons in the energy range from 250 to 2000 eV at a high flux of up to 10^13^ photons s^–1^. Further details regarding the design and working principle of the HIPPIE beamline and endstation can be found in Zhu et al.^30^ In our work, the analysis chamber was coupled to the catalysis cell to specifically study solid samples in the presence of a gaseous atmosphere. This cell was isolated from the ultrahigh vacuum (UHV) environment of the analysis chamber. Gases of specific interest are introduced into the cell through a gas inlet, allowing the study of solid–gas interfaces. The cell pressure can be conditioned over a wide range from UHV (10^–8^ mbar) to 30 mbar, enabling surface investigations under atmospherically relevant temperature and pressure conditions. In this study, all experiments were carried out with the sample at 16 °C.

### 2.2 Sample Preparation and Surface Measurements

(NH_4_)_2_SO_4_ (Sigma-Aldrich, ≥99.0%) was dissolved in deionized water (Milli-Q) to form a solution. Surface samples used for APXPS measurements were prepared by pipetting the (NH_4_)_2_SO_4_ solution onto gold-coated sample plates. The samples were heated to (≈60 °C) to dry and formed a thin layer of (NH_4_)_2_SO_4_ crystals. The sample plate is later transferred to a sample carousel within the storage chamber of the APXPS endstation before analysis. The unique design of the sample carousel enables six samples to be prepared and stored simultaneously. During measurements, individual samples are transferred from the storage chamber to the analysis chamber, where gas-phase GL was introduced by evaporating a 40 wt % GL–water solution (CAS number: 107-22-2, Merck) from the evaporator. The total pressure of water and GL mixture in our measurements varies from 1.89 to 2.35 mbar. We assumed the reported pressure is predominantly attributed to water vapor. This is because water vapor dominates the pressure in GL–water solutions due to its higher evaporation rate and the formation of GL oligomers with low volatility while drying.^31,32^ In addition, a viscous GL–water solution was observed in the evaporator at the end of the evaporation, further supporting our assumption. Thus, the RH within the cell is estimated based on the assumption that the pressure resulted solely from water vapor, making this estimate an upper limit for the actual RH. To change the RH during experiments, the GL–water gas pressure was regulated by a leak valve while the temperature was kept constant at 16 °C. In the control measurements, helium (He) was dosed, and the (NH_4_)_2_SO_4_ surface was considered as a pure reference sample for comparison with the GL-exposed (NH_4_)_2_SO_4_ surface. The pressure of He within the analysis chamber was maintained at 3.4–4.8 mbar. This elevated pressure was employed to mitigate the charging effect,^33^ which works as photoionized electrons emitted from the gas phase can compensate for the otherwise inherent buildup of positive charge on the surface of nonconductive samples. The charging effect artificially reduces the kinetic energy (KE) of photoelectrons. Figure S1 shows an example of the sample surface placed within the analysis chamber alongside the electron analyzer cone as captured during one measurement. The pressures and temperatures measured during our experiments and the derived RH are provided in Table S1. Two additional atmospherically relevant salt samples, sodium sulfate (Na_2_SO_4_) and sodium chloride (NaCl), were also prepared following the same procedure, and their surface properties were investigated.

Depth profiles of elements in (NH_4_)_2_SO_4_ samples were acquired at three kinetic energy (KE) levels: 350, 500, and 750 eV, corresponding to mean escape depths (MED) of 1.09 1.49, and 1.87 nm, respectively. MED^34^ represents the average distance that photoelectrons travel without experiencing inelastic scattering losses at a given KE. In addition, the oxygen K-edge NEXAFS spectra of He- and GL-exposed (NH_4_)_2_SO_4_ surface were acquired under different RH conditions by scanning the KE from 525 to 560 eV. This energy range was selected to avoid photoemission peaks while scanning at the absorption threshold. Beam damage of the samples initially manifested as a decay of the detected signal over time. This was mitigated using the exit slits of the beamline to control the photon flux incident on the sample and by regularly moving the sample to probe fresh material.

### 2.3 Data Analysis

All recorded photoemission spectra from APXPS measurements were aligned using the binding energy (BE) of aliphatic carbon as a reference (C 1s at 284.8 eV). The resulting BEs of electrons from different atoms and orbitals in (NH_4_)_2_SO_4_, including oxygen 1s (O 1s, 531.8 eV), nitrogen 1s (N 1s, 401.8 eV), sulfur 2p (S 2p, 167.8 eV), and carbon 1s (C 1s, 284.8 eV), are listed in Table S2. After a constant background was subtracted, the XPS spectra were fitted with Gaussian functions using Igor Pro software (version 8) to identify potential chemical components. To identify the observed XPS peaks of specific chemical components, we performed core electron binding energy (BE) calculations of potential products on weakly hydrated (NH_4_)_2_SO_4_ using snapshots extracted from first-principle molecular dynamics (MD) simulations, following the strategy outlined in Kong et al.^35^ and described in full detail in Text S2. The MED of photoelectrons in (NH_4_)_2_SO_4_ are calculated by QUASES-IMFP-TPP2M version 3,^36^ based on the relationship between MED of photoelectrons for most elements and their KEs.

## 3 Results and Discussion

### 3.1 Surface Elemental Composition

**Figure 1:**
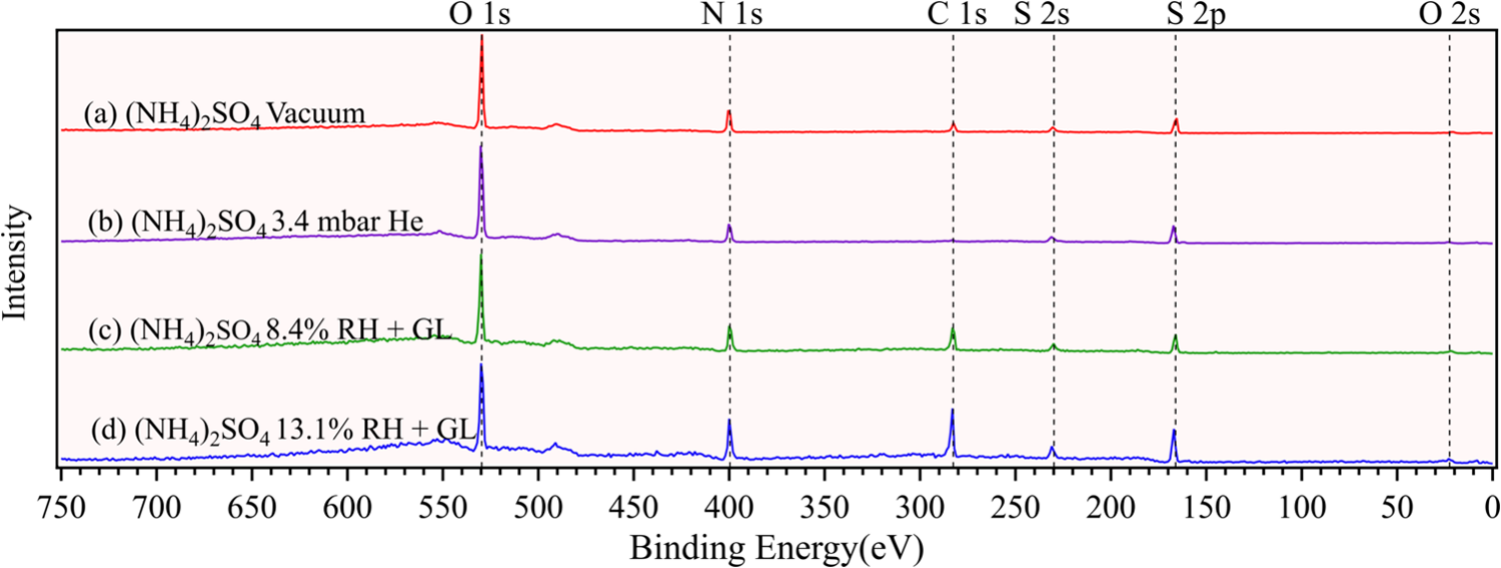
Surveys of (NH_4_)_2_SO_4_ exposed to He and a mixture of GL and water. The surveys were acquired at a photon energy of 1000 eV. All peaks in the surveys were normalized based on the height of the O 1s peak. The vapor pressure of He is indicated in the figure. The RH of the GL–water mixtures is also shown and is calculated by assuming the reported pressure is attributable solely to water vapor. The vapor pressures of GL–water mixtures are provided in Table S1.

The broadband photoemission spectra (referred to as surveys hereafter) of He- and GL-exposed (NH_4_)_2_SO_4_ surfaces were collected at a photon energy (PE) of 1000 eV, as shown in Figure 1. The surface composition of each sample was characterized by resolving the peaks corresponding to the BEs of photoelectrons emitted from different atoms. All peaks in surveys were normalized based on the height of oxygen 1s (O 1s) peak. As shown in the photoemission spectra (Figure 1), elements of interest include O 1s, nitrogen 1s (N 1s), carbon 1s (C 1s), and sulfur 2s and 2p (S 2s and S 2p). All expected elements from pure dry (NH_4_)_2_SO_4_ (N 1s, O 1s, S 2p) were identified (Figure 1a,b), with their BEs listed in Table S1. A C 1s peak appears in pure (NH_4_)_2_SO_4_ sample under the vacuum condition (Figure 1a), indicating the presence of adventitious carbon (C–C Adv.), likely due to chamber contamination, residues from prior measurements, or sample preparation processes, as noted in previous research.^37^ No C 1s peak is observed for the He-exposed (NH_4_)_2_SO_4_ sample (Figure 1b), indicating no detectable carbon contamination under the He atmosphere.

Upon introducing GL–water vapor at RH = 8.4 and 13.1%, the XPS spectra of (NH_4_)_2_SO_4_ (Figure 1c,d) exhibit distinct features compared to those in the background measurement (Figure 1a,b). A significant and broad C 1s peak is observed in surveys collected under different vapor pressure conditions (Figure 1c,d), indicating the presence of detectable organic compounds on the (NH_4_)_2_SO_4_ surface. Notably, when GL–water vapor was introduced, the intensities of the N 1s, C 1s, and S 2p peaks relative to the O 1s peak at RH = 8.4 and 13.1% (Figure 1c,d) were comparable to or even higher than those observed under vacuum/He conditions (Figure 1a,b). This is unexpected because an increase in the level of water introduction would lead to greater water surface adsorption, but our observations reveal the opposite trend. Our results suggest that nitrogen and sulfur in (NH_4_)_2_SO_4_ are likely interacting with nonoxygen elements from GL, such as carbon, leading to the formation of new chemical species. This finding is further discussed in the following sections. The XPS spectra of Na_2_SO_4_ and NaCl are also examined, with results shown in Figure S2 and discussed in Text S1 in detail. In general, the identified BEs for all atoms in the three salts examined ((NH_4_)_2_SO_4_, Na_2_SO_4_, and NaCl) are consistent with those reported in previous studies,^35,38^ confirming appropriate sample preparation and effective performance of the APXPS.

### 3.2 Nitrogen-Containing Organic Compounds Formed on GL-Reacted (NH_4_)_2_SO_4_ Surface

**Figure 2:**
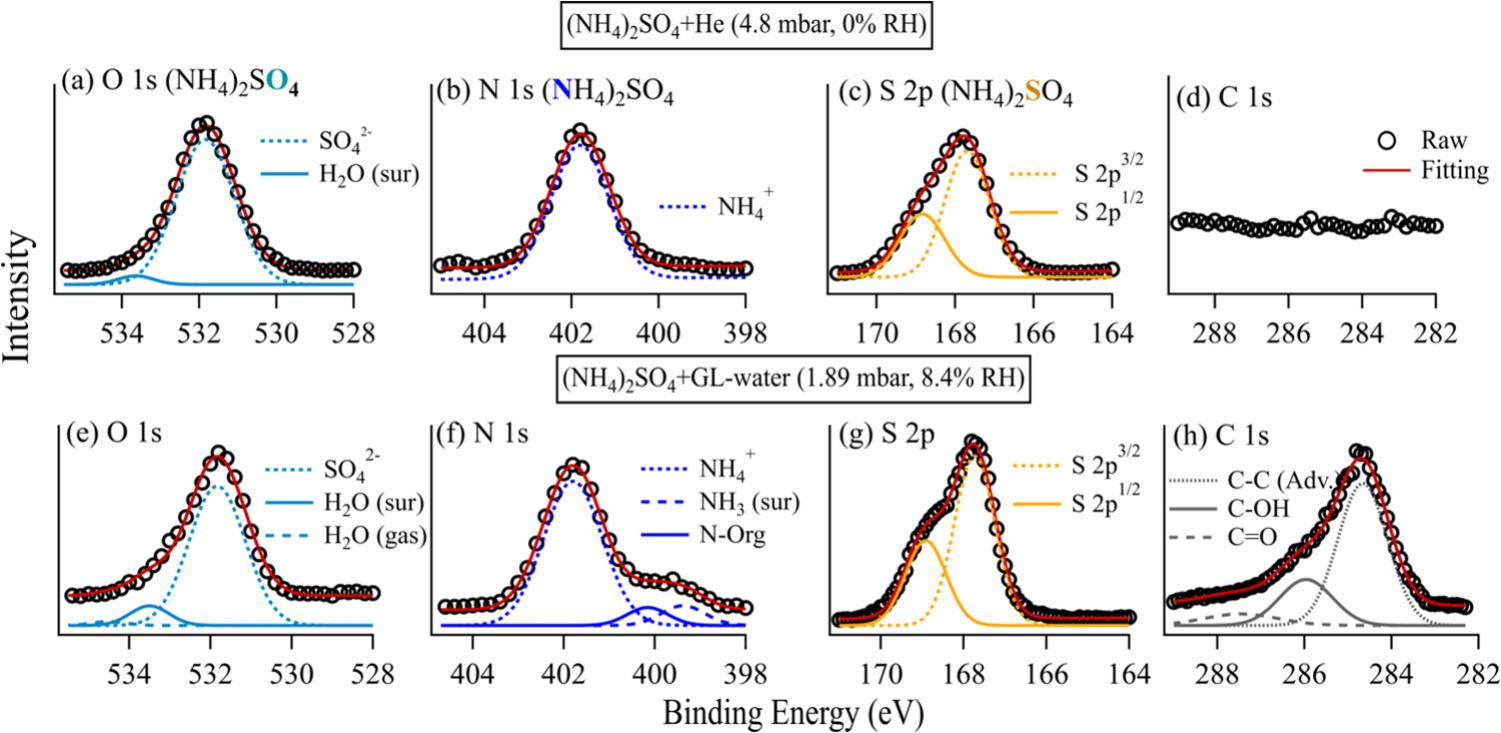
XPS spectra measured on an (NH_4_)_2_SO_4_ surface exposed to (a–d) He and (e–h) GL–water atmospheres, respectively. The XPS spectra were obtained at the edge of (a, e) oxygen 1s (O 1s, PE: 887 eV), (b, f) nitrogen 1s (N 1s, PE: 1150 eV), (c, g) sulfur 2p (S 2p, PE: 1000 eV), and (d, h) carbon 1s (C 1s, PE: 1040 eV). The partial pressures of the He and GL–water mixtures are 4.8 and 1.89 mbar, respectively, which correspond to RH = 0 and 8.4% (Table S1).

Figure 2 shows XPS spectra of individual elements obtained on an (NH_4_)_2_SO_4_ surface exposed to He (Figure 2a,d) and the GL–water vapor at RH = 8.4% (Figure 2e–h). The XPS spectra of (NH_4_)_2_SO_4_ obtained under other RH conditions (11.2 and 13.3%) are shown in Figure S3: as the spectral features at RH = 11.2 and 13.3% are similar to those observed at RH = 8.4%, they are not discussed further. XPS spectra were acquired at the edges of O 1s (Figure 2a,e), N 1s (Figure 2b,f), S 2p (Figure 2c,g), and C 1s (Figure 2d,h). The chemical compositions resolved from each XPS spectrum are shown in Figure 2, with their BEs detailed in Table S3.

For the He-exposed (NH_4_)_2_SO_4_ sample (Figure 2a–d), the O 1s spectrum (Figure 2a) is primarily from the absorption by SO_4_^2–^, with a minor contribution from surface water, H_2_O(sur). As noted in our previous study,^35^ H_2_O(sur) is very strongly bonded with salt surfaces and is not completely removed from the drop-cast samples through heating and evaporation. N 1s and S 2p spectra of pure (NH_4_)_2_SO_4_ (Figure 1b,c) are well represented by single species (i.e., NH_4_^+^ and SO_4_^2–^, respectively) and their BEs are consistent with previous results^35^ reported for (NH_4_)_2_SO_4_ sample at RH = 3%. Notably, the S 2p spectrum exhibits doublet peaks, S 2p^3/2^ at ≈167.6 eV and S 2p^1/2^ at ≈168.8 eV. The S 2p spin–orbit splitting of ≈1.2 eV matches the value reported by Fauré et al.^39^ for magnesium sulfate (MgSO_4_). No significant carbon signal has been detected for the He-exposed (NH_4_)_2_SO_4_ sample (Figure 2d), indicating no carbon contamination from the sample and environment. When helium is introduced, it competes with adventitious carbon for interactions with the surface, preventing carbon deposition.

The fit of the O 1s spectrum for GL-exposed (NH_4_)_2_SO_4_ (Figure 2e) reveals the presence of three oxygen-containing species: H_2_O(gas), H_2_O(sur), and SO_4_^2–^. The S 2p spectra for both He-exposed (Figure 2c) and GL-exposed (NH_4_)_2_SO_4_ surfaces (Figure 2g) show identical doublet peaks, indicating that sulfur remained stable in terms of its oxidation state and other properties that could influence its BE.

In the N 1s spectrum for GL-exposed (NH_4_)_2_SO_4_ (Figure 2f), two additional nitrogen-containing species are present compared to pure (NH_4_)_2_SO_4_ (Figure 2b): nitrogen in surface-bound NH_3_ at a BE of 399.3 eV^35^ (NH_3_(sur)) and nitrogen in organics at a BE of 400.1 eV (BE of C–N bond). The formation of NH_3_(sur) is well supported, as the difference of BE of NH_3_(sur) relative to NH_4_^+^ (ΔBE = 2.5 eV), obtained in our experiment (Figure 2f), is comparable to that observed by Kong et al.^35^ (ΔBE = 2.6 eV). The formation of NH_3_(sur) on pure (NH_4_)_2_SO_4_ surface is also reported in other studies^35,40^ and attributed to the deprotonation of NH_4_^+^. The observed C–N bond suggests the formation of nitrogen-containing organic compounds (N-Org) through interactions between GL and the (NH_4_)_2_SO_4_ surface. This observation also aligns with previous chamber experiments,^24−26,28^ suggesting that N-Org are common SOA products formed through GL uptake on both wet and dry (NH_4_)_2_SO_4_ aerosol.

The C 1s fit (Figure 2h) shows the formation of carbon-containing species on the (NH_4_)_2_SO_4_ surface upon introduction of GL, including C–C (BE at 284.6 eV), C–OH (BE at 286.0 eV), and C=O (BE at 287.5 eV). The C=O peak is associated with GL adsorbed on the (NH_4_)_2_SO_4_ surface, and the C–C peak is assigned to adventitious carbon (C–C Adv.). The C–OH peak might indicate the formation of GL oligomers as a result of GL hydration.^32^ Although newly formed N-Org is observed in the N 1s spectrum, the C–N bond cannot be distinguished in the C 1s spectrum. This is due to the difficulty in differentiating the carbon in N-Org from that in C–OH and C=O species,^41^ given its low concentration and the complexity of the carbon spectrum.^41^ One previous chamber study^28^ proposed organosulfate formation via reactive uptake of GL on (NH_4_)_2_SO_4_ aerosol particles, by observing fragments of GL-sulfates using AMS. However, sulfur-containing carbon (C–S) was not identifiable in our C 1s fit (Figure 2h). This is consistent with the S 2p fit (Figure 2g) of GL-exposed (NH_4_)_2_SO_4_ showing similar spectral features to that of the pure (NH_4_)_2_SO_4_ sample (Figure 2c), indicating no formation of new sulfur-containing species distinguishable from SO_4_^2–^. It is challenging to conclude whether organosulfate was formed on the GL-(NH_4_)_2_SO_4_ surface based on our APXPS measurements, given that undetectable organosulfate formation might be attributed to difficulties in distinguishing organosulfate from inorganic sulfate or from other organic compounds in XPS spectra. For example, the SO_4_^2–^ absorption in organosulfate could overlap with that of (NH_4_)_2_SO_4_ in the S 2p spectrum due to the same oxidation state of sulfur within them (Figure 2g). Additionally, as shown by Moulder and Chastain,^41^ C–S exhibits absorption over a wide BE range (from 285.5 to 287.5 eV), which overlaps with absorption attributed to C–OH (BE = 286.0 eV), further complicating precise identification.

### 3.3 Imines Identified as Potential N-Org Products

**Figure 3:**
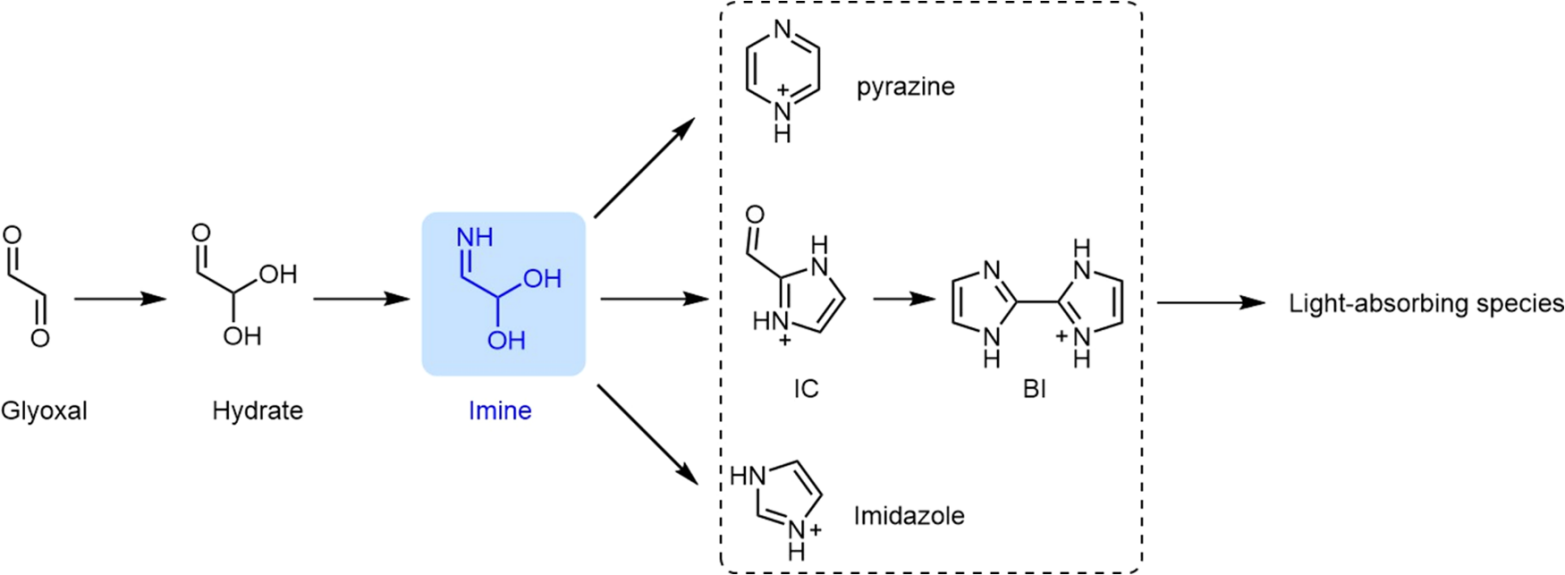
Transformation pathways of GL on (NH_4_)_2_SO_4_ particle surfaces under RH = 5% condition, proposed by De Haan et al.^27^ based on results from their aerosol chamber experiments and from previous studies.^22^ Pyrazine, biimidazole (BI), imidazole-2-carboxaldehyde (IC), and imidazole (dashed box) are products detected by De Haan et al.^27^ using AMS. Imine (blue) is newly detected by our APXPS measurements.

The APXPS results suggest surface chemical changes when (NH_4_)_2_SO_4_ is exposed to GL, while previous chamber studies investigated the same system from a different perspective, focusing on reaction pathways and product formation within the aerosol bulk. Based on results reported by chamber experiments, De Haan et al.^27^ summarized the potential reaction pathways of GL-(NH_4_)_2_SO_4_ aerosol (RH = 5%), a simplified view of which is presented in Figure 3. Notably, imine was proposed as a crucial intermediate in the formation of light-absorbing organic species, yet it was not directly detected in previous studies. Instead, particle-phase imidazole, pyrazine, and IC were observed by AMS as key products transformed from imines during GL-(NH_4_)_2_SO_4_ reactions.^27^

To further identify the N-Org detected in our study, we employed core electron BE calculations for potential products interacting with a weakly hydrated (NH_4_)_2_SO_4_ surface. The considered products included imine, imidazole, pyrazine, IC and IB. The simulated chemical interactions between potential organic products and the (NH_4_)_2_SO_4_ surface are visualized in Figure S4. The computed BE of nitrogen in different organic products and its difference (ΔBE) relative to that of nitrogen in (NH_4_)_2_SO_4_ are detailed in Table 1. As shown in the N 1s spectrum of the GL-exposed (NH_4_)_2_SO_4_ surface (Figure 2f), the detected ΔBE of nitrogen in N-Org and in (NH_4_)_2_SO_4_ is −1.6 eV. A similar ΔBE value was found in the simulation with imine (Figure S4a, −1.3 eV in Table 1), implying that the observed N-Org product is most likely imine. ΔBE value simulated with other species (imidazole, pyrazine, IC, and IB) ranges from −0.9 ± 0.2 to 1.5 ± 0.2 eV. Imine is recognized as an essential intermediate in GL-(NH_4_)_2_SO_4_ surface reactions (Figure 3).^27^ However, detecting it in situ is challenging due to its rapid transformation and the limited resolution of available probing techniques, making its identification particularly significant. We cannot rule out the formation of other N-Org compounds such as pyrazine, IC, and imidazole, as the detected N-Org spectrum may represent a combined contribution from multiple products. However, simulations of ΔBE of different products indicate that imine exhibits the closest ΔBE to the observed value, indicating its relatively significant contribution compared with other potential products. Additionally, we cannot exclude the formation of compounds that have not been detected or characterized in prior chamber studies and have been simulated in our work. Further simulations focusing on interactions between (NH_4_)_2_SO_4_ surface and other potential organic compounds are needed.

**Table 1:** Core Electron BE of N 1s Lines for Five N-Org Species (Imine, Imidazole, Pyrazine, IC, and BI) Interacting with the (NH_4_)_2_SO_4_ Surface, Calculated from Molecular Dynamics Snapshots^a^

specie	specie atom name	ΔBE (eV)
(NH_4_)_2_SO_4_ crystal	N	0
imine	NI^b^	–1.3 ± 0.1
imidazole	ND	0.5 ± 0.1
pyrazine	N3, N4	–0.9 ± 0.2
IC	N1, N2	–0.5 ± 0.2
IB	N1	1.5 ± 0.2
	N2	1.4 ± 0.2
	N3	–0.8 ± 0.2
	N4	0.7 ± 0.2

a BEs are reported relative (ΔBE) to the N 1s of the nitrogen atom in (NH_4_)_2_SO_4_. Uncertainty presents a single standard deviation, derived as described in Text S2.

b NI, ND, N1, N2, N3, and N4 are different nitrogen atoms in various N-Org, as shown in Figure S4.

### 3.4 Elemental Ratio Depth Profiles for GL-Exposed (NH_4_)_2_SO_4_ Surfaces

**Figure 4:**
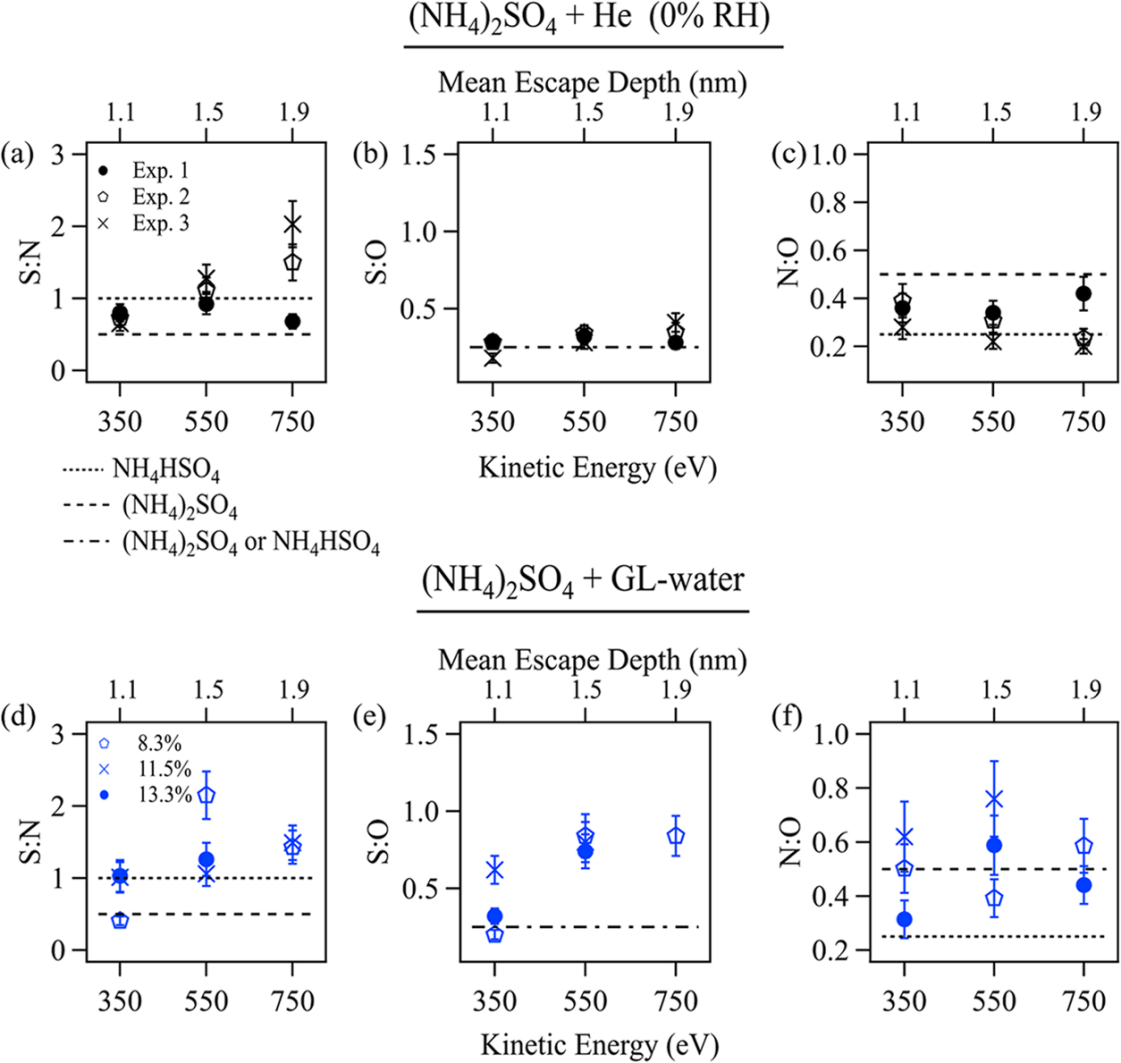
Depth profile of elemental ratios on (NH_4_)_2_SO_4_ surface exposed to (a–c) He and to (d–f) GL–water. The KEs 350, 500, and 750 eV correspond to MEDs of 1.1, 1.5, and 1.9 nm, respectively. Experiments 1–3 in (a)–(c) represent repeated experiments on (NH_4_)_2_SO_4_ surface under a He atmosphere. Different symbols in (d)–(f) represent experiments on the GL-exposed (NH_4_)_2_SO_4_ surface under different gas pressure/RH conditions. The elemental ratios of S/N, S/O, and N/O of reference anhydrous (NH_4_)_2_SO_4_ and NH_4_HSO_4_ are indicated with the dashed lines with values of 0.5 and 1 (S/N), 0.25 and 0.25 (S/O), and 0.5 and 0.25 (N/O). The error bars represent the uncertainties associated with the elemental ratios arising from spectral fitting and instrumental errors (Text S3).

Figure 4 presents the elemental ratios of selected elements as a function of KE on the He-exposed (Figure 4a–c) and GL-exposed (NH_4_)_2_SO_4_ surface (Figure 4d–i) under various RH conditions (0, 8.3, 11.5, and 13.3%). The MED of photoelectrons (1.1, 1.5, and 1.9 nm) corresponding to each KE (350, 550, and 750 eV) is displayed on the upper *x*-axes. The uncertainties associated with the determined elemental ratios are indicated by error bars shown in Figure 4, which are derived based on spectral fitting uncertainty and instrumental errors. More details on the uncertainty calculation are provided in Text S3, and the relative errors determined for each elemental ratio are listed in Table S4. The theoretical elemental ratios of S/N, S/O, and N/O on pure (NH_4_)_2_SO_4_ and NH_4_HSO_4_ surfaces are indicated by the dashed lines.

Figure 4a–c shows the elemental ratio results probed on He-(NH_4_)_2_SO_4_ surfaces and demonstrates generally good agreement among repeated experiments (Exp. 1–3). As shown in Figure 4a, the S/N ratios obtained on He-(NH_4_)_2_SO_4_ surfaces exceed 0.5, which is a reference value of the S/N ratio for pure anhydrous (NH_4_)_2_SO_4_. This implies that the measured surface is unlikely to present as pure (NH_4_)_2_SO_4_, rather it is likely mixed with other components yielding a higher S/N ratio, for example, pure NH_4_HSO_4_ with a S/N ratio of 1. This finding is further supported by the detected S/O and N/O ratios on He-(NH_4_)_2_SO_4_ surfaces (Figure 4b,c), which are close to 0.25, matching the reference values for pure NH_4_HSO_4_. The elemental depth profile on the (NH_4_)_2_SO_4_ surface obtained here is consistent with those observed by Kong et al.^35^ using APXPS. They demonstrated that the (NH_4_)_2_SO_4_ surface presented as a mixture of (NH_4_)_2_SO_4_ and NH_4_HSO_4_ at RH = 3%.

Upon the introduction of GL, the elemental ratios obtained on the (NH_4_)_2_SO_4_ surface shifted, showing greater variability across experiments under different pressure conditions (Figure 4d–f). An increase in S/O and N/O ratios was observed on GL-exposed (NH_4_)_2_SO_4_ compared to pure (NH_4_)_2_SO_4_ surface (Figure 4e,f vs 4b,c). The increased N/O ratios, to values above 0.5, are attributed to the formation of N-Org with higher nitrogen content than NH_4_HSO_4_ (N/O = 0.25), such as imine (N/O = 0.5) or other N-Org components (pyrazine, IC, and imidazole). Similarly, a significant increase in the S/O ratios (S/O = 0.5–0.8, Figure 4e) was observed for GL-exposed surface compared to pure (NH_4_)_2_SO_4_/NH_4_HSO_4_ samples (S/O = 0.25, Figure 4b). This suggests the formation of sulfur-containing organics (S-Org) with a lower oxygen content compared to SO_4_^2–^. The increased N/O and S/O values in GL-exposed samples align with the findings from surveys (Figure 1d), indicating that nitrogen and sulfur in (NH_4_)_2_SO_4_ preferentially bond with carbon from GL rather than with oxygen from water on its surface, which leads to a relative reduction of oxygen compared to nitrogen and sulfur. As shown in S 2p (Figure 2g) and C 1s XPS spectra (Figure 2h) and discussed above, it is difficult to distinguish the formed S-Org from inorganic sulfate (SO_4_^2–^) in S 2p (Figure 2g) and from other organic compounds in C 1s fits (Figure 2h). Therefore, unlike for N-Org, the exact chemical species of S-Org formed on the GL-reacted surface cannot be unambiguously identified. Our findings demonstrate that the formed S-Org cannot be solely attributed to organosulfate, as the observed S/O ratios exceed that of SO_4_^2–^. The majority of S/N values (Figure 4d) are similar to those of pure (NH_4_)_2_SO_4_ (Figure 4a), indicating that only a small fraction of the nitrogen and sulfur on the (NH_4_)_2_SO_4_ surface bonds with GL to form N-Org and S-Org. As a result, the sample surface maintains the characteristic S/N ratio of NH_4_HSO_4_/(NH_4_)_2_SO_4_. This also explains why it is challenging to distinguish S-Org and N-Org from other components given their low concentrations relative to the (NH_4_)_2_SO_4_ bulk surface.

The elemental ratios of the formed N-Org with other elements (N, O, and S) under different RH conditions are shown in Figure S5. The derived N-Org ratios are associated with significant error bars (Figure S5 and Table S4), highlighting the uncertainties in quantifying surface-bound N-Org. At higher RHs (11.5 and 13.3%), the N-Org/N ratio is slightly greater than at RH = 8.4% (Figure S5a), indicating that N-Org formation may be favored by higher concentrations of GL and water vapor. However, this increase becomes less significant when the uncertainties associated with elemental ratios are considered. Furthermore, no clear trend was observed for the N-Org/N ratios depending on MED (Figure S5a–c), suggesting no obvious surface depletion or enrichment of N-Org relative to NH_3_ and NH_4_^+^ species. Similar to the N-Org/N ratio, no distinct MED-dependent trend was observed for the N-Org/S and N-Org/O ratios.

### 3.5 GL-Reacted (NH_4_)_2_SO_4_ Surface Changes at Low-RH Conditions

**Figure 5:**
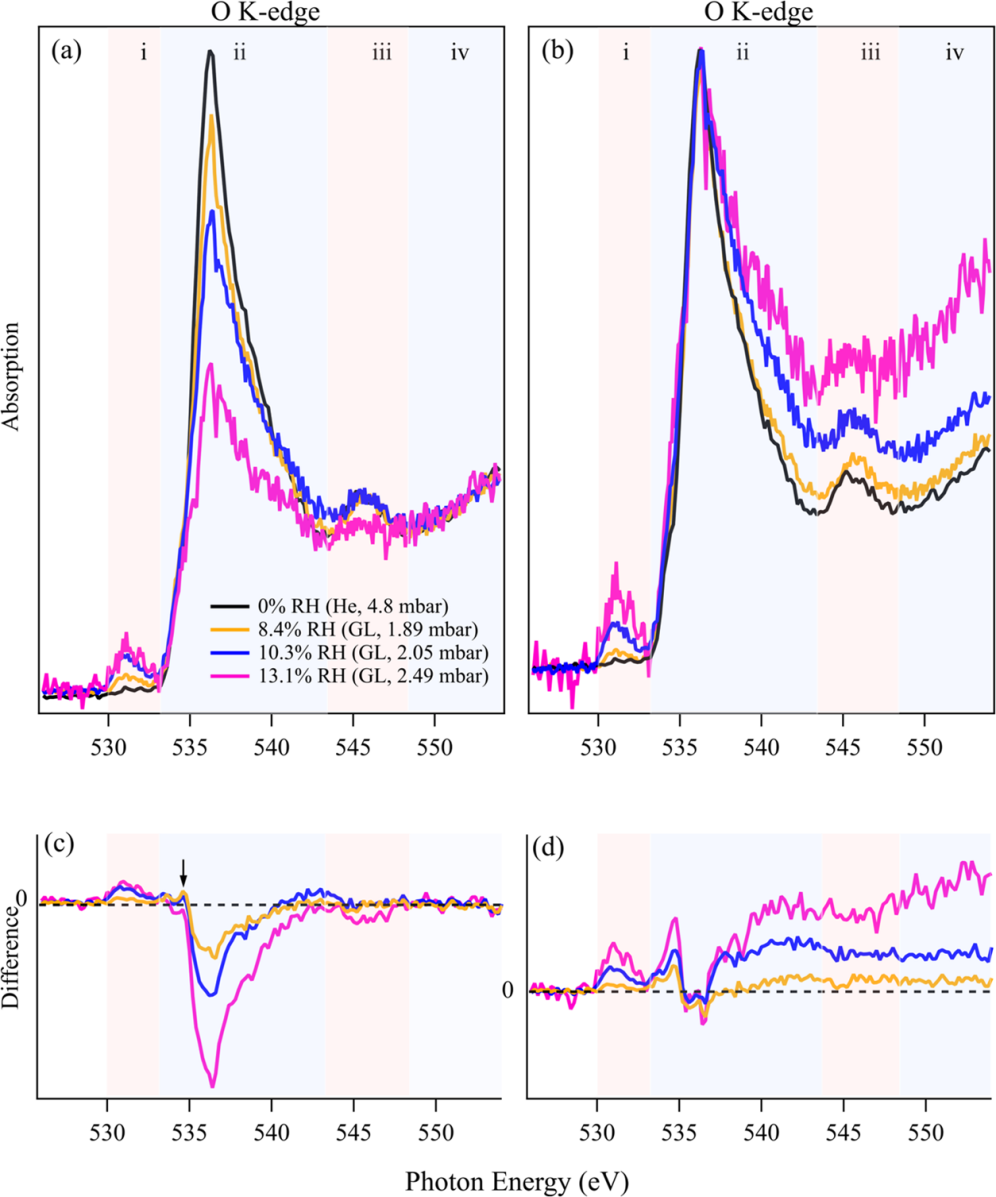
NEXAFS spectra at oxygen K-edge of (NH_4_)_2_SO_4_ surface exposed to He (black lines) and GL–water, normalized to (a) pre- and post-edge, and (b) pre-edge and peak signal at 536.2 eV. (c, d) Difference spectra between the GL-exposed (NH_4_)_2_SO_4_ spectra and the reference He-exposed (NH_4_)_2_SO_4_ spectrum derived from (a) and (b), respectively. The vapor pressure of the He or GL–water mixture and the RH are marked on each panel. Four features of the oxygen K-edge spectra are identified: region (i) 529.7–533.1 eV; (ii) 533.1–543.3 eV; (iii) 543.3–548.4 eV; and the post-edge region (iv) 548.4–555.0 eV.

The GL-exposed (NH_4_)_2_SO_4_ surfaces under changing RH conditions (8.4, 10.3, and 13.1%) were characterized by NEXAFS at the oxygen K-edge. The obtained spectra were normalized to the pre- and post-edge (Figure 5a), as well as to the pre-edge and the peak signal at 536.2 eV (Figure 5b), respectively. As control experiments, the O–K-edge spectrum of the He-exposed (NH_4_)_2_SO_4_ surface was also acquired (black line in Figure 5a,b). Four spectral features were identified in the O–K-edge (NH_4_)_2_SO_4_ spectra (Figure 5a,b): region (i) 529.7–533.1 eV; (ii) 533.1–543.3 eV; (iii) 543.3–548.4 eV; and the post-edge region (iv) 548.4–555.0 eV.

For He-exposed (NH_4_)_2_SO_4_ surfaces (black lines in Figure 5a,b), two prominent absorption peaks in regions (ii) and (iii) were observed, resembling the spectral features of dry (NH_4_)_2_SO_4_ particles, as reported by Zelenay et al.^42^ Specifically, the absorption peak at 536.2 eV in region (ii) was assigned to the transition from O 1s to an S–O antibonding orbital in sulfate.^39,43^ Upon exposure to GL–water, an absorption peak appeared in region (i) of all GL-exposed (NH_4_)_2_SO_4_ spectra (Figure 5a,b), which was absent in the He-exposed (NH_4_)_2_SO_4_ spectrum (black line). This feature corresponds to the 1s → π* transitions of oxygen in carbonyl-type functional groups (C=O)^44^ or oxygen in nitrate,^45^ indicating the presence of GL or production of other C=O-containing compounds. This absorption (region (i)) increases with gas pressure, as expected, due to an increased partial pressure of GL that enhances the surface adsorption of GL and facilitates the formation of other C=O-containing compounds.

In addition to the change in region (i), a decline in absorption was observed in region (ii) for all GL-exposed samples (Figure 5a,b). To better understand the mechanism underlying this decline, absorption differences between GL-exposed and He-exposed (NH_4_)_2_SO_4_ surfaces, depending on photon energy, were calculated. The resulting difference spectra are presented in Figure 5c,d, where the He-exposed (NH_4_)_2_SO_4_ spectrum was used as the baseline and subtracted from the GL-exposed (NH_4_)_2_SO_4_ spectra. Negative values in difference spectra indicate reduced oxygen absorption on the (NH_4_)_2_SO_4_ surface following GL exposure and vice versa. As shown in Figure 5c, a significant reduction in absorption was observed at 534.6–542.4 eV in region (ii). The distribution of the absorption difference aligns with the prominent absorption peak in region (ii) of the He-exposed (NH_4_)_2_SO_4_ surface (black line, Figure 5a), as well as the sulfate absorption at 533–538 eV reported in pure (NH_4_)_2_SO_4_^42^ and MgSO_4_ samples.^39^ This suggests that the reduction in absorption in region (ii) is due to the decreased Auger electron yield from sulfate absorption. This reduction could result from either the screening effects caused by an adsorbed water layer on the (NH_4_)_2_SO_4_ surface or the dilution effects of a solvated surface. This interpretation is further supported by the fact that the magnitude of this reduction increases with RH, indicating that sulfate absorption diminishes with higher GL and water exposure.

The difference spectra derived from O K-edge spectra normalized to the pre-edge and sulfate peak at 536.2 eV (Figure 5b) are shown in Figure 5d. Substantial absorption enhancement was observed at 535.0 eV (region (ii)). This feature aligns with the absorption of free-hydrogen bond in liquid water at 535.0 eV, as suggested by previous studies.^46,47^ The increased absorption at this energy region (Figure 5c) demonstrates that water condenses on the (NH_4_)_2_SO_4_ surface when GL–water vapor was introduced. This enhancement (feature at 535.0 eV) grows with the vapor pressure and aligns with the observations in Figure 5a, reinforcing the conclusion that water condensation is promoted by greater GL–water exposure. This finding is also supported by the minor peak observed at 534.6 eV in Figure 5c normalized to pre- and post-edge (black arrow), indicating water condenses on the (NH_4_)_2_SO_4_ surface upon GL–water exposure. Combined with the above discussion of Figure 5c, it suggests that the observed reduction in sulfate absorption is at least partially caused by condensed water on (NH_4_)_2_SO_4_ surfaces. An enhanced absorption was observed at 536.8–543.6 eV in region (iii) (Figure 5d), consistent with observations in MgSO_4_ samples reported by Fauré et al.^39^ who attributed this absorption to adsorbed water on the sample surface. This observation again indicates that water condenses on the (NH_4_)_2_SO_4_ surface upon exposure to a mixture of GL and water vapor. The absorption in post-edge region (iv) in Figure 5b represents the total oxygen of the sample surface within the probed energy range. When the spectra (Figure 5b,5d) are normalized to the sulfate peak (536.2 eV), the increased absorption difference in region (iv) of Figure 5d indicates increased total oxygen in the GL-exposed (NH_4_)_2_SO_4_ surface, despite the reduced oxygen absorption by sulfate under higher gas pressure conditions (Figure 5c). This increase is instead attributed to the increased relative contribution of oxygen from nonsulfate sources, such as water and GL. Our findings from O K-edge NEXAFS results normalized to different signals demonstrate that GL–water exposure of (NH_4_)_2_SO_4_ leads to a reduction in sulfate absorption and is likely due to the adsorption of water on (NH_4_)_2_SO_4_ surfaces.

The NEXAFS spectra for the (NH_4_)_2_SO_4_ N K-edge were also acquired and are shown in Figure S6. The spectrum of the GL-exposed (NH_4_)_2_SO_4_ sample is similar to that of He-exposed (NH_4_)_2_SO_4_, with a main spectral feature identified at 405–410 eV corresponding to nitrogen in (NH_4_)_2_SO_4_. Additional details about the N K-edge of (NH_4_)_2_SO_4_ can be found in Text S4 but are not discussed here, due to the low quality attributed to the influence of the silicon nitride (Si_3_N_4_) X-ray window.

The oxygen NEXAFS measurements indicate that GL and water condense onto (NH_4_)_2_SO_4_ surfaces under low-RH conditions (RH ≤ 13.3%). The condensation was expected to lead to higher total oxygen content of the (NH_4_)_2_SO_4_ surface. However, increased elemental ratios of both S/O and N/O at high GL–water pressures observed by APXPS measurements and depth profiles demonstrate that oxygen is relatively reduced. Together the results from APXPS and NEXAFS are only explained by the formation of low-oxygen N-Org and S-Org species on the GL-exposed (NH_4_)_2_SO_4_ surface, as otherwise the S/O and N/O ratios would increase. In general, our surface characterization of (NH_4_)_2_SO_4_ indicates that GL–water exposure leads to surface reactions on (NH_4_)_2_SO_4_ surfaces yielding atmospherically relevant organic species.

## 4 Conclusions

This study demonstrates that GL undergoes surface reactions on dry (NH_4_)_2_SO_4_ particles at a low RH ≤ 13.3%. Using APXPS and NEXAFS spectroscopy, N-Org and S-Org compounds, associated with the formation of BrC, were identified on GL-exposed (NH_4_)_2_SO_4_ surfaces. The comparison between experimentally determined BE and calculated BE, from first-principle molecular dynamics simulations for N-Org species, suggests that imines are the most likely N-Org species formed. In contrast, the S-Org species could not be distinguished based on BE, which is not significantly affected by surface chemistry. Depth profiling of elemental ratios on (NH_4_)_2_SO_4_ surfaces indicates that GL exposure leads to notable changes in the surface composition. Increased S/O and N/O ratios were observed on GL-exposed (NH_4_)_2_SO_4_ compared to He-exposed (NH_4_)_2_SO_4_, suggesting that nitrogen and sulfur in (NH_4_)_2_SO_4_ interact with carbon rather than oxygen from GL, forming N-Org and S-Org with low oxygen content. No clear trend was observed in the ratio of N-Org to other elements (N, O, and S) as a function of MED under varying RH conditions, indicating that N-Org is uniformly distributed across the probed topmost surface depth. Oxygen NEXAFS measurements confirm the presence of organics and water adsorbed on (NH_4_)_2_SO_4_ surfaces when GL and water were introduced, which is consistent with the XPS findings. These findings provide compelling evidence of surface-driven N-Org and S-Org formation pathways on ammonium salt particles, which constitutes atmospheric BrC. Given the critical role of BrC in air quality and global climate, future studies should examine such surface-specific organic formation pathways on a broader range of aerosol particle species reacting with diverse organic gas precursors to better assess their atmospheric relevance.

## Supporting Information Available

Photo of the analysis chamber; additional XPS spectra; computational details of the first-principles molecular dynamics (FPMD) simulations and core BE calculations; uncertainties of elemental ratios; and nitrogen K-edge NEXAFS (PDF)
