# Correction to “Surface Formation Pathway of Nitrogen- and Sulfur-Containing Organic Compounds on Ammonium Sulfate”

**DOI:** 10.1021/acs.jpca.5c02055

**Published:** 2025-04-10

**Authors:** Jie Chen, George Wandera Kisimbiri, Ivan Gladich, Nicolas Fauré, Erik S. Thomson, Robert Temperton, Zamin A. Kanji, Xiangrui Kong

The Acknowledgment section incorrectly stated the beamline used. The correct beamline is **HIPPIE beamline at MAX IV Laboratory**.

