# Supplementary material for: Surface Formation Pathway of Nitrogen- and Sulfur-Containing Organic Compounds on Ammonium Sulfate
Source: J Phys Chem A. 2025 Mar 13;129(12):2922–31. doi: 10.1021/acs.jpca.5c00332 (PMC11956130; doi:10.1021/acs.jpca.5c00332)
Supplement: Supplementary file 1 — jp5c00332_si_001.pdf [file jp5c00332_si_001.pdf]

Supporting Information for

Surface Formation Pathway of Nitrogen and Sulfur Containing Organic Compounds on Ammonium Sulfate

Jie Chen[1,#], George Wandera Kisimbiri[2,#], Ivan Gladich[3,4], Nicolas Fauré[2], Erik S. Thomson[2], Robert Temperton[5], Zamin A. Kanji[1,*], Xiangrui Kong[2,*]

1 Institute for Atmospheric and Climate Science, ETH Zürich, Zurich, 8092, Switzerland

2 Department of Chemistry and Molecular Biology, University of Gothenburg, SE-413 90 Gothenburg, Sweden

3 European Centre for Living Technology (ECLT), Dorsoduro, Calle Crosera, 30124 Venice, Italy

4 Qatar Environment and Energy Research Institute, Hamad Bin Khalifa University, P.O. Box 31110, Doha, Qatar

5 MAX IV Laboratory, Lund University, SE221-00 Lund, Sweden

Correspondence to: xiangrui.kong@chem.gu.se and zamin.kanji@env.ethz.ch

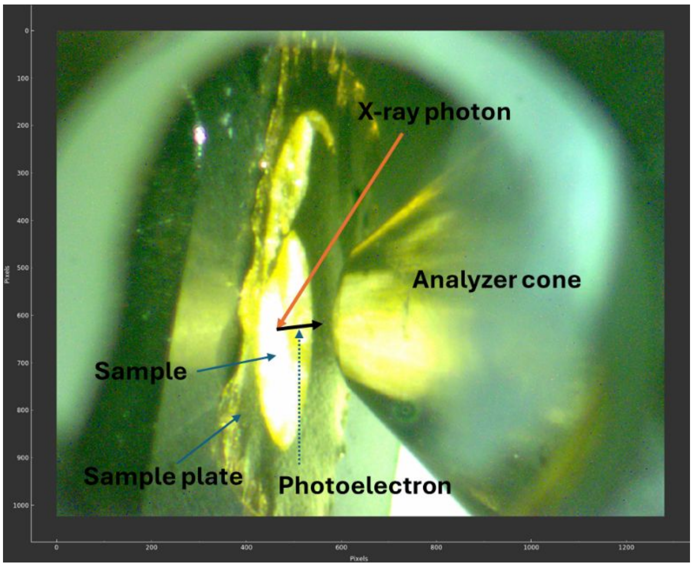

Figure S1. Images of one sample surface inside the analysis chamber as taken during a beamtime measurement at the MAX IV synchrotron laboratory.

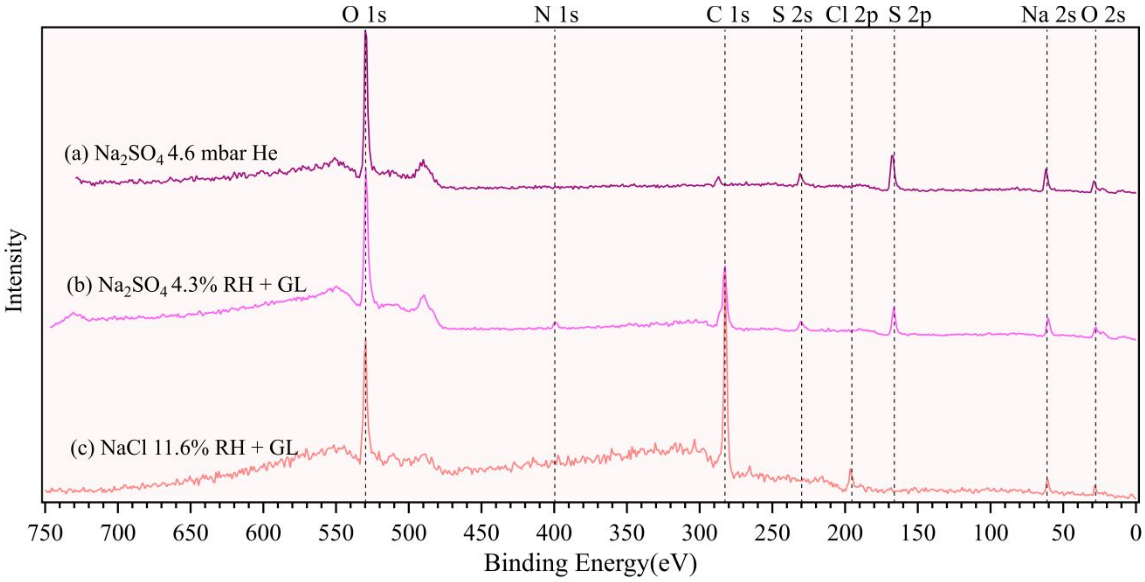

Figure S2. Surveys of Na_2SO_4 (a-b) and NaCl (c) exposed to He and a mixture of GL and water. The surveys were acquired at a photon energy of 1000 eV. The partial pressure of He is 4.6 mbar. The RH of the GL-water mixture in each experiment is also shown, which is calculated by assuming the reported pressure is predominantly attributed to water vapor, also listed in Table S1.

Text S1. All expected elements from Na_2SO_4 (Na 2s, S 2p, S 2s, O 1s) and NaCl (Na 2s, Cl 2p) were identified, with their binding energies (BEs) listed in Table S2. In the GL-exposed Na_2SO_4 sample (Figure S2b), a minor N 1s peak is observed, possibly resulting from chamber contamination, such as residual

$(NH_4)_2SO_4$ from prior measurements. The C 1s peak observed in the survey of He-exposed Na_2SO_4 (Figure S2a) is also due to chamber contamination. The Na 2s signal in the GL-exposed NaCl sample is not significant (Figure S2g), likely because of its lower photoionization cross-section[1] compared to other elements (e.g., O 1s and C 1s) at the applied PE (1000 eV). In general, there is good agreement between the obtained XPS spectra for Na_2SO_4 and NaCl in this study and those reported in our previous study,[1] indicating the good performance of the present APXPS measurements.

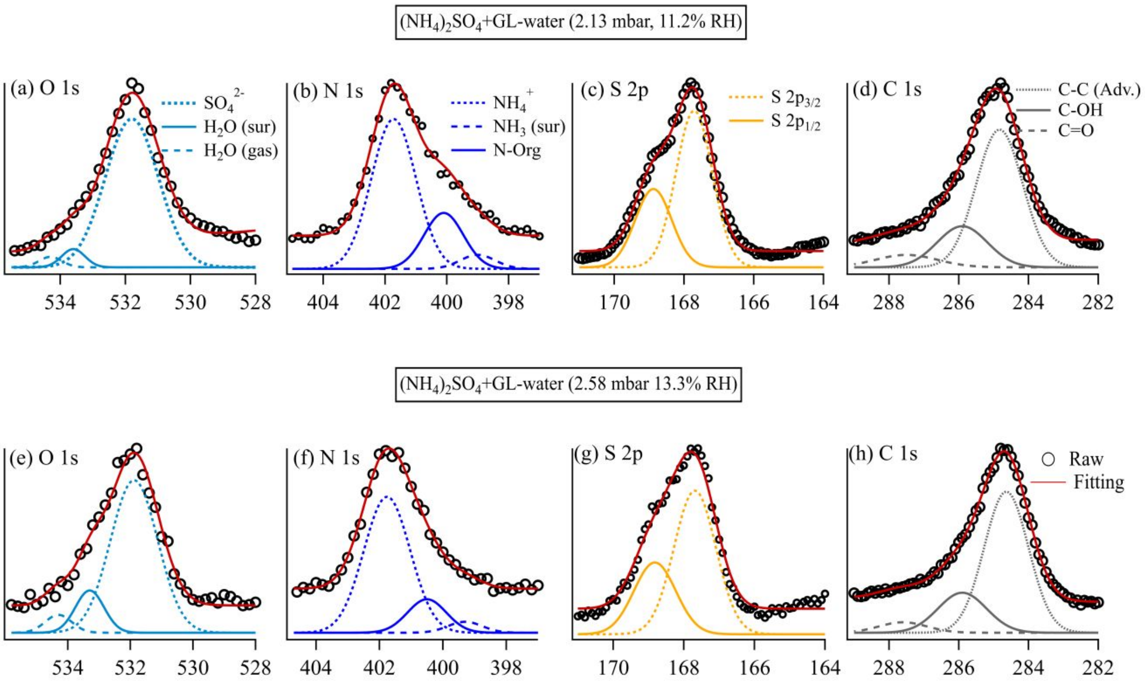

Figure S3. XPS spectra of $(NH_4)_2SO_4$ exposed to a mixture of glyoxal and water (GL-water) at RH = 11.5% (a-d) and RH = 13.3% (e-h). The XPS spectra were obtained at the edge of (a, e) oxygen 1s (O 1s, PE: 887 eV), (b, f) nitrogen 1s (N 1s, PE: 1150 eV), (c, g) sulfur 2p (S 2p, PE: 1000 eV) and (d, h) carbon 1s (C 1s, PE: 1040 eV). The partial pressure of the GL-water mixture is 2.13 mbar and 2.58 mbar (see Table S1), which corresponds to a RH of 11.2% and 13.3%, respectively, assuming only contribution from water vapor.

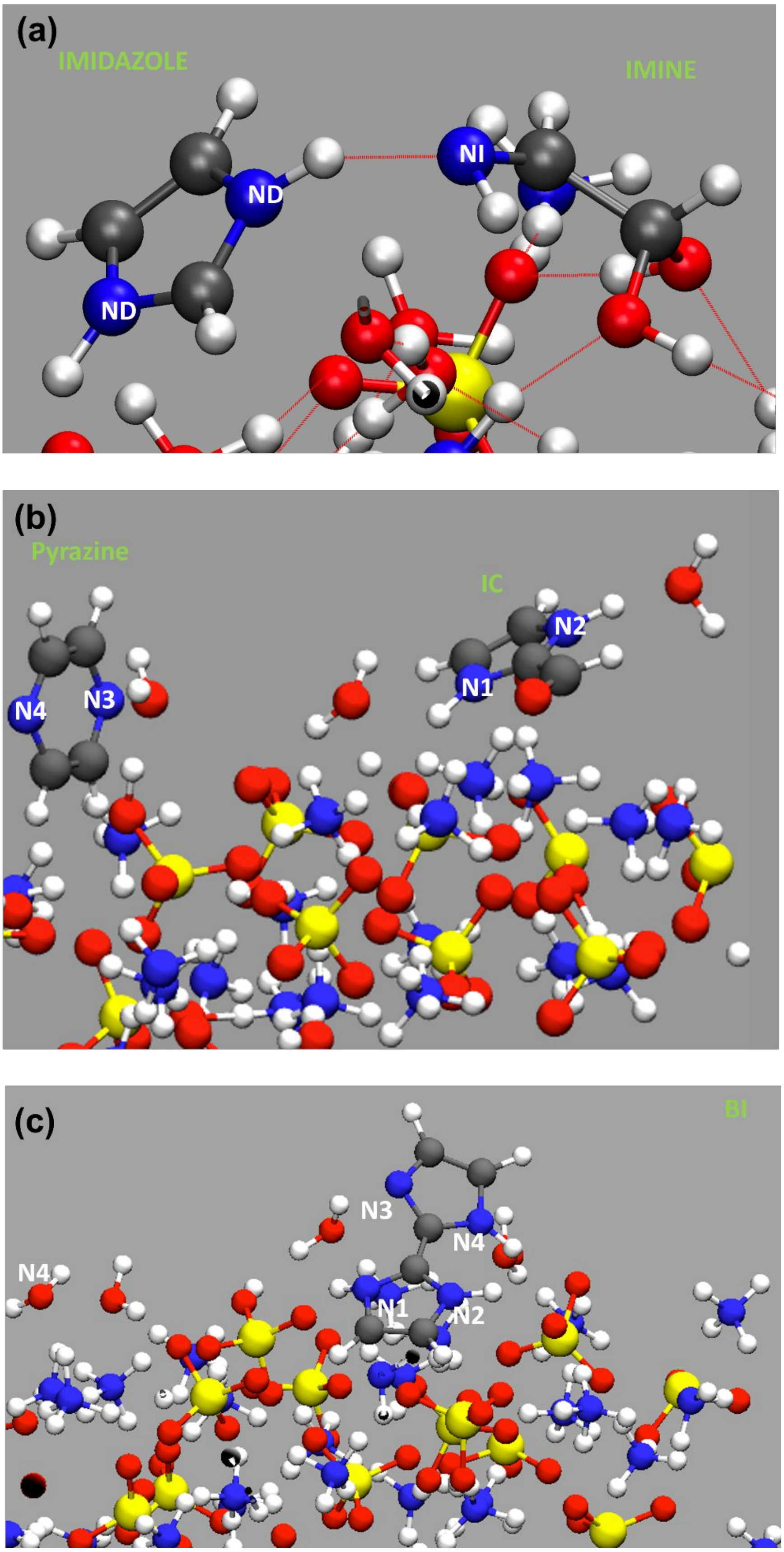

Figure S4. Simulated chemical interaction between five species, including (a) imidazole and imine, (b) pyrazine and IC, and (c) BI, and glyoxal on a weakly hydrated $(NH_4)_2SO_4$ surface. The simulated core

electron binding energy (BE) of nitrogen atoms in these 5 species and the difference (ΔBE) compared to those in $(NH_4)_2SO_4$ are listed in Table 1.

Text S2. Computational details of the first-principles Molecular Dynamics (FPMD) simulations and core binding energy (BE) calculations.

The computational procedure for caulating the N 1s lines of Imine, Imidazole, pyrazine, IC, and IB on the surface of a weakly hydrated $(NH_4)_2SO_4$ surface closely resemble our previous approach implemented in Kong et al. [2] An initial $(NH_4)_2SO_4$ crystal, consisting of a single (bi-)layer containing 16 SO_4^{2-} and 32 NH_4^+ ions, was constructed based on the structure reported by Schlemper and Hamilton [3]. The initial structure, with dimensions of 1.5 nm, 2.1 nm, and 0.6 nm along the X, Y, and Z axes, respectively, underwent geometry optimization for relaxation. Subsequently, the Z-dimension was extended to 3.6 nm, creating a simulation box featuring two crystal/vacuum interfaces. Finally, the system was further optimized with 8 water molecules that were placed on one crystal/vacuum interface, resembling a weakly hydrated crystal surface (i.e., about one adsorbed water sublayer, ≈2.5 water molecules/nm^2). Strating from this configuration three different systems were created, as shown in Figure S4:

1) $(NH_4)_2SO_4$ with 8 water, 1 Imine, and 1 Imidazole (Figure S4a)

2) $(NH_4)_2SO_4$ with 8 water, 1 Pyrazine, and 1 IC (Figure S4b)

3) $(NH_4)_2SO_4$ with 8 water, 1 IB (Figure S4c)

For each system we performed three independent 40 ps constant volume and temperature (NVT) molecular dynamics (MD) simulations at first-principle level, i.e., calculating forces on-the-fly by density functional theory (DFT). Here, First-principles molecular dynamics (FPMD) simulations were carried out using the PBE functional[4, 5] with Grimme's D3 dispersion correction[6] and a DZVP basis set. The basis set was truncated with a 500 Ry energy cutoff. Valence electrons were explicitly included, while core electrons were modeled using pseudopotentials[7]. The temperature was maintained at 300 K using an adaptive Langevin thermostat[8] with a time constant of 300 fs.

Experimental XPS data were compared with theoretically calculated Core Electron Binding Energies (BEs). According to Koopmans' theorem,[9, 10] BEs are defined as the negative of the orbital energies corresponding to the peaks in the Density of States (DOS). It is known that the absolute positions of DOS peaks may deviate from experimental values due to approximations in DFT, such as electron correlation, relativistic effects, and the neglect of orbital relaxation.[10-14] Nevertheless, despite these limitations, the relative positions of DOS peaks within the same element, referred to as chemical shifts, generally align well with experimental shifts across many chemical systems[10]. Due to its simplicity, DOS provides a computationally efficient method for calculating chemical shifts and interpreting experimental XPS spectra. This approach is particularly advantageous in soft matter studies[15-19] (or, as in this case, for adsorbed water on crystal surfaces) where the adsorbants can explore different strutural configurations and, thus, statistically meaningful BEs need to be derived from multiple system snapshopts.

Density of States (DOS) calculations were performed using all-electron computations with the PBE-D3 functional[4, 5], employing the Gaussian Augmented Plane Wave (GAPW) method as implemented in CP2K[20]. The Pcseg-3 basis set,[11, 14] specifically designed for core ionization studies in conjunction with DFT, was utilized alongside the PBE functional. The DOS calculations were performed with over 40 snapshots extracted at 1 ps intervals from the 40 ps FPMD trajectory, for a total of 120 computed DOS over 120 FPMD frames (i.e., 40 snapshots for each of the three NVT runs). Following the methodology

in Kong et al. [2] and Pham et al. [15], the calculated DOS were smoothed using a Gaussian broadening with a width of 0.5 eV. The average BE and associated uncertainty for the N1s lines were determined by applying a weighted average to the smeared Gaussian spectral intensities of the N1s peaks. This approach was chosen because each DOS represents an independent measurement of the system at different time points, ensuring a statistically robust representation of the system's behavior over the trajectory.

All geometry optimizations, FPMD and DOS calculations were performed using the CP2K molecular dynamics package[21].

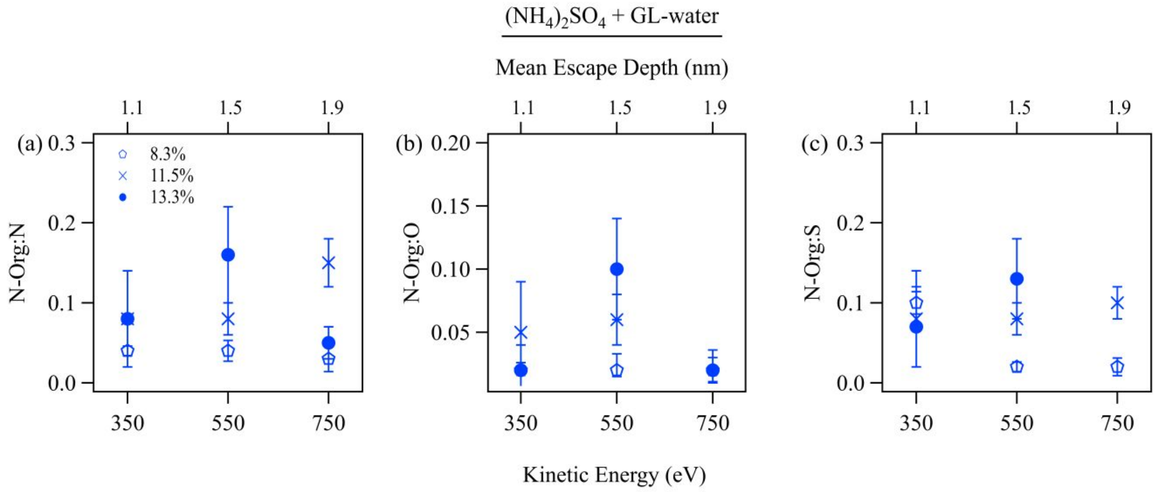

Figure S5. The depth profiles for elemental ratios of the formed N-Org with other elements (N, O and S) under different RH conditions (8.3%, 11.5% and 13.3%). Kinetic energies of 350, 500 and 750 eV correspond to mean escape depths (MEDs) of photoelectrons of 1.1, 1.5 and 1.9 nm, respectively. The RH indicated was estimated based on the assumption that the pressure resulted solely from water vapor, making this estimate an upper limit for the actual RH.

Text S3. Calculate uncertainties of elemental ratios. The uncertainties associated with the determined elemental ratios account for errors arising from XPS spectra fitting and instrumental errors. The XPS spectra were fitted with Gaussian functions using Igor Pro Software Version 8 to identify the detected chemical components. The error arising from fitting the area of a spectrum is provided by the software, and the relative error can be calculated by dividing the fitting error by the total signal ($E_{fitting}$). The instrumental error is considered to be **10%** of the total signal ($E_{instrument}$), based on studies conducted by an expert from MAX VI Laboratory (Dr. Robert Temperton). Therefore, the relative error for each spectrum corresponding to an individual element is the combination of these two sources of error ($E_{element} = E_{fitting} + E_{instrument}$). The relative error for the elemental ratio is derived using the error summation error propagation. For example, if the relative error of sulfur (S, E_s) and oxygen (O, E_o) are known, the relative error for the S:O ratio ($E_{S:O}$) can be calculated using the following equation:

$$E_{S:O} = \sqrt{{E_S}^2 + {E_O}^2}$$

The derived relative errors of elemental ratios are listed in Table S5.

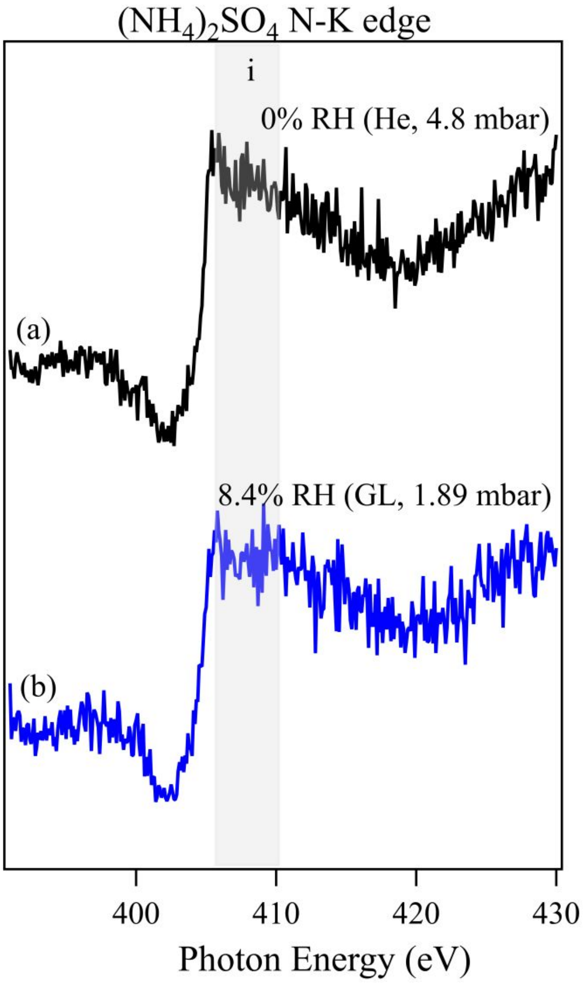

Figure S6. Nitrogen K-edge NEXAFS spectra for $(NH_4)_2SO_4$ surface exposed to helium (a, He) and glyoxal and water (b, GL). One feature of the nitrogen K-edge spectra is identified: region (i) 405.0–410.0 eV, which is assigned to the nitrogen in $(NH_4)_2SO_4$. The deep valley in the N K-edge spectrum at 402 eV is attributed to X-ray absorption by the silicon nitride (Si_3N_4) window.

Text S4. The main peak at PE of 405 eV to 410 eV in the N K-edge spectra (Figure S6a and S6b) is assigned to the nitrogen in $(NH_4)_2SO_4$, corresponding to 1s→σ* transition as suggested by Leinweber et al. [22] No significant changes to the N K-edge spectrum have been observed when increasing RH from 0% to 8.4%, indicating that the introduction of GL and water vapor did not significantly change nitrogen in $(NH_4)_2SO_4$. This observation might seem inconsistent with the N-Org formation detected by APXPS measurements on GL-reacted $(NH_4)_2SO_4$ at RH≤13.3%. However, this discrepancy is likely due to the low concentration of nitrogen in N-Org products relative to the abundance of nitrogen in the $(NH_4)_2SO_4$ surface, making it difficult to detect N-Org formation using NEXAFS. Additionally, the N K-edge spectra are of lower quality due to the influence of the silicon nitride (Si_3N_4) X-ray window, which absorbs photons in the critical energy range (402 eV), further complicating detection. Further NEXAFS measurements focusing on Nitrogen on GL-reacted $(NH_4)_2SO_4$ surface are therefore recommended.

Table S1. The measured temperatures (T) and pressures (P) and the calculated relative humidities (RH s) during the experiments. The P_{eq} is the equilibrium water vapor pressure at the measured temperature, and the P_{act} refers to the actual water vapor pressure that was measured after dosing.

RH (%)	T (°C)	P_{eq} (mbar)	P_{act} (mbar)
13.8	16.88	19.22	2.65
13.3	17.00	19.36	2.58
13.1	17.00	19.36	2.54
12.8	16.99	19.35	2.48
11.5	17.01	19.37	2.23
11.2	17.03	19.40	2.13
10.2	19.30	22.37	2.29
9.9	19.50	22.65	2.25
8.4	19.30	22.37	1.89
8.3	19.50	22.65	1.89
7.9	19.50	22.65	1.80
4.3	20.10	23.51	1.00

Table S2. The binding energies (BEs) for different atoms and the corresponding chemical composition in $(NH_4)_2SO_4$, Na_2SO_4 and NaCl.

**$(NH_4)_2SO_4$**	**Atom**	**N 1s**	**S 2p**	**C 1s**	**O 1s**
	**BE (eV)**	401.8	167.8	284.8	531.8
	**Species**	NH_4^+	SO_4^{2-}	Aliphatic	SO_4^{2-}
**Na_2SO_4**	**Atom**	**Na 2s**	**S 2p**	**C 1s**	**O 1s**
	**BE (eV)**	63.5	167.8	284.8	531.8
	**Species**	Na+	SO_4^{2-}	Aliphatic	SO_4^{2-}
**NaCl**	**Atom**	**Na 2s**	**Cl 2p**	**C 1s**	
	**BE (eV)**	63.5	190	284.8	
	**Species**	Na+	Cl-	Aliphatic	

Table S3. The binding energies (BEs) for the resolved chemical composition and functional groups on surfaces of helium and glyoxal-exposed $(NH_4)_2SO_4$, as shown in Figure 1.

Edge	BE (eV)	Species
N 1s	401.8	NH_4^+
	400.1	nitrogen-containing organics
	399.3	NH_3(sur)
S 2p	167.8	S $2p^{3/2}$
	167.6	S $2p^{1/2}$
O 1s	531.8	SO_4^{2-}
	533.6	surface H_2O
	534.3	gaseous H_2O
C 1s	284.6	adventitious C-C
	286.0	C-OH
	287.5	C=O

Table S4. The relative errors of derived elemental ratios depending on the mean escape depth (MED) of photoelectrons.

			$(NH_4)_2SO_4$+He_EXP1			
MED (nm)	S:N	S:O	N:O			
1.1	16%	15%	16%			
1.5	15%	15%	15%			
1.9	15%	15%	16%			
			$(NH_4)_2SO_4$+He_EXP2			
MED (nm)	S:N	S:O	N:O			
1.1	18%	15%	19%			
1.5	16%	15%	17%			
1.9	16%	16%	18%			
			$(NH_4)_2SO_4$+He_EXP3			
MED (nm)	S:N	S:O	N:O			
1.1	16%	15%	16%			
1.5	15%	15%	16%			
1.9	16%	16%	16%			
			$(NH_4)_2SO_4$+GL_8.3% RH			
MED (nm)	S:N	S:O	N:O	N-Org:S	N-Org:N	N-Org:O
1.1	16%	16%	17%	14%	16%	16%
1.5	15%	16%	17%	29%	29%	30%
1.9	16%	16%	17%	45%	46%	46%
			$(NH_4)_2SO_4$+GL_11.5% RH			
MED (nm)	S:N	S:O	N:O	N-Org:S	N-Org:N	N-Org:O
1.1	20%	14%	20%	77%	79%	77%
1.5	16%	17%	18%	24%	25%	26%
1.9	16%	-	-	21%	22%	-
			$(NH_4)_2SO_4$+GL_13.3% RH			
MED (nm)	S:N	S:O	N:O	N-Org:S	N-Org:N	N-Org:O
1.1	21%	16%	23%	77%	78%	77%
1.5	18%	15%	19%	37%	39%	38%
1.9	-	-	17%	-	33%	33%

The “-” denotes experiments in which an element was not detected, and thus, the elemental ratio and its relative error were not determined.

Reference

(1) Kong, X.; Gladich, I.; Fauré, N.; Thomson, E. S.; Chen, J.; Artiglia, L.; Ammann, M.; Bartels-Rausch, T.; Kanji, Z. A.; Pettersson, J. B. C. Adsorbed Water Promotes Chemically Active Environments on the Surface of Sodium Chloride. *The Journal of Physical Chemistry Letters* **2023**, *14* (26), 6151-6156. DOI: 10.1021/acs.jpclett.3c00980.

(2) Kong, X.; Castarède, D.; Thomson, E. S.; Boucly, A.; Artiglia, L.; Ammann, M.; Gladich, I.; Pettersson, J. B. C. A surface-promoted redox reaction occurs spontaneously on solvating inorganic aerosol surfaces. *Science* **2021**, *374* (6568), 747-752. DOI: 10.1126/science.abc5311.

(3) Schlemper, E. O.; Hamilton, W. C. Neutron-Diffraction Study of the Structures of Ferroelectric and Paraelectric Ammonium Sulfate. *The Journal of Chemical Physics* **1966**, *44* (12), 4498-4509. DOI: 10.1063/1.1726666.

(4) Perdew, J. P.; Ruzsinszky, A.; Csonka, G. I.; Vydrov, O. A.; Scuseria, G. E.; Constantin, L. A.; Zhou, X.; Burke, K. Restoring the Density-Gradient Expansion for Exchange in Solids and Surfaces. *Physical Review Letters* **2008**, *100* (13), 136406. DOI: 10.1103/PhysRevLett.100.136406.

(5) Perdew, J. P.; Burke, K.; Ernzerhof, M. Generalized Gradient Approximation Made Simple. *Physical Review Letters* **1996**, *77* (18), 3865-3868. DOI: 10.1103/PhysRevLett.77.3865.

(6) Grimme, S.; Antony, J.; Ehrlich, S.; Krieg, H. A consistent and accurate ab initio parametrization of density functional dispersion correction (DFT-D) for the 94 elements H-Pu. *Journal of Chemical Physics* **2010**, *132* (15), 154104. DOI: 10.1063/1.3382344.

(7) Goedecker, S.; Teter, M.; Hutter, J. Separable dual-space Gaussian pseudopotentials. *Physical Review B* **1996**, *54* (3), 1703-1710. DOI: 10.1103/PhysRevB.54.1703.

(8) Jones, A.; Leimkuhler, B. Adaptive stochastic methods for sampling driven molecular systems. *The Journal of Chemical Physics* **2011**, *135* (8). DOI: 10.1063/1.3626941 (acccessed 12/11/2024).

(9) Szabo, A.; Ostlund, N. S. *Modern quantum chemistry: introduction to advanced electronic structure theory*; Courier Corporation, 1996.

(10) Tardio, S.; Cumpson, P. J. Practical estimation of XPS binding energies using widely available quantum chemistry software. *Surface and Interface Analysis* **2018**, *50* (1), 5-12. DOI: 10.1002/sia.6319.

(11) Ambroise, M. A.; Jensen, F. Probing Basis Set Requirements for Calculating Core Ionization and Core Excitation Spectroscopy by the Δ Self-Consistent-Field Approach. *Journal of Chemical Theory and Computation* **2019**, *15* (1), 325-337. DOI: 10.1021/acs.jctc.8b01071.

(12) Kahk, J. M.; Lischner, J. Core electron binding energies of adsorbates on Cu(111) from first-principles calculations. *Physical Chemistry Chemical Physics* **2018**, *20* (48), 30403-30411. DOI: 10.1039/C8CP04955F.

(13) Golze, D.; Keller, L.; Rinke, P. Accurate Absolute and Relative Core-Level Binding Energies from GW. *The Journal of Physical Chemistry Letters* **2020**, *11* (5), 1840-1847. DOI: 10.1021/acs.jpclett.9b03423.

(14) Jensen, F. How Large is the Elephant in the Density Functional Theory Room? *The Journal of Physical Chemistry A* **2017**, *121* (32), 6104-6107. DOI: 10.1021/acs.jpca.7b04760.

(15) Pham, T. A.; Govoni, M.; Seidel, R.; Bradforth, S. E.; Schwegler, E.; Galli, G. Electronic structure of aqueous solutions: Bridging the gap between theory and experiments. *Science Advances* **2017**, *3* (6), e1603210. DOI: 10.1126/sciadv.1603210.

(16) Gaiduk, A. P.; Govoni, M.; Seidel, R.; Skone, J. H.; Winter, B.; Galli, G. Photoelectron Spectra of Aqueous Solutions from First Principles. *Journal of the American Chemical Society* **2016**, *138* (22), 6912-6915. DOI: 10.1021/jacs.6b00225.

(17) Ping, Y.; Li, Y.; Gygi, F.; Galli, G. Tungsten Oxide Clathrates for Water Oxidation: A First Principles Study. *Chemistry of Materials* **2012**, *24* (21), 4252-4260. DOI: 10.1021/cm3032225.

(18) Theodorakopoulos, G.; Csizmadia, I. G.; Robb, M. A.; Kucsman, Á.; Kapovits, I. Experimental (ESCA) and theoretical (SCF–MO) determination of the oxidation state of sulphur in bis(2-carboxyphenyl) sulphur dihydroxide dilactone. *Journal of the Chemical Society, Faraday Transactions* **1977**, *73*, 293-297. DOI: 10.1039/F29777300293.

(19) Giesbers, M.; Marcelis, A. T. M.; Zuilhof, H. Simulation of XPS C1s Spectra of Organic Monolayers by Quantum Chemical Methods. *Langmuir* **2013**, *29* (15), 4782-4788. DOI: 10.1021/la400445s.

(20) Ferrario, M.; Ciccotti, G.; Binder, K. *Computer Simulation in Condensed Matter: From Materials to Chemical Biology. Vol. 2*; Springer, 2006.

(21) Hutter, J.; Iannuzzi, M.; Schiffmann, F.; VandeVondele, J. cp2k: atomistic simulations of condensed matter systems. *WIREs Computational Molecular Science* **2014**, *4* (1), 15-25. DOI: 10.1002/wcms.1159.

(22) Leinweber, P.; Kruse, J.; Walley, F. L.; Gillespie, A.; Eckhardt, K.-U.; Blyth, R. I. R.; Regier, T. Nitrogen K-edge XANES - an overview of reference compounds used to identify `unknown' organic nitrogen in environmental samples. *Journal of Synchrotron Radiation* **2007**, *14* (6), 500-511. DOI: 10.1107/S0909049507042513.
